# Characterisation of tree vibrations based on the model of orthogonal oscillations

**DOI:** 10.1038/s41598-018-26726-5

**Published:** 2018-06-04

**Authors:** Ivana Kovacic, Dragi Radomirovic, Miodrag Zukovic, Benka Pavel, Milutin Nikolic

**Affiliations:** 1 University of Novi Sad, Faculty of Technical Sciences, 21000 Novi Sad, Serbia; 2 University of Novi Sad, Faculty of Agriculture, 21000 Novi Sad, Serbia

## Abstract

This study presents quantitative and qualitative insights into the analysis of data obtained by tracking the motion of reflective markers arranged along the trunk of a pole-like potted tree, which was recorded by a state-of-the-art infrared motion-tracking system. The experimental results showed in-plane damped trajectories of the markers with lateral displacements, i.e. out-of-plane vibrations of the tree under consideration. To explain such response and to determine the corresponding oscillatory characteristics, a completely new and original utilisation of the recorded in-plane damped trajectories is presented. The quantitative insight gained is based on the mechanical model that consists of two orthogonal springs and dampers placed in the plane where the motion takes place, and it is then directed towards the determination of the characteristics of the related orthogonal oscillations: two natural frequencies, the position of the principal axes to which they correspond, and two damping ratios. The qualitative insight gained involves analysing the shape and narrowness of the trajectory to assess how close-valued two natural frequencies are, and how small the overall damping is. The quantitative and qualitative methodologies presented herein are seen as beneficial for arboriculture, forestry and botany, but given the fact that orthogonal oscillations appears in many natural and engineering systems, they are also expected to be useful for specialists in other fields of science and engineering as well.

## Introduction

New experimental techniques are seen as beneficial for getting enhanced insights and deeper understanding of trees’ oscillatory characteristics. Different motion-capture systems can be particularly promising, which, for example, includes tree motion sensors^1^. A high-tech motion-capture system is used in this project for recording tree vibrations, although it was originally developed for studying human motion. However, no matter whether one uses contemporary or traditional experimental techniques, these characteristics need to be correctly defined and determined. This is especially important for a natural frequency, since it is seen as the indicator for the conditions leading to tree uprooting^2,3^, which can cause property damage and personal injury or even death. Different approaches have been used to obtain natural frequencies from the motion in the time domain (displacement-time diagrams) or the response in the frequency domain^4^. It is of interest here to investigate which oscillatory features and parameters can be determined if one focuses on the trajectories of free motion. This is motivated by the fact that the literature review revealed a shortage of information available for the trajectories of motion, as well as the fact that the trajectories are usually shown just to illustrate the full complexity of the tree response in two dimensions^5^. Peltola *et al*.^6^ measured wind and resulting swaying of two Scots pines (*Pinus sylvestris*), illustrating graphically that the trajectory in one plane formed by the east-west direction and the north-south direction implied swaying mainly perpendicular to the direction of mean wind speed. Moore and Maguire^7^ investigated Douglas-fir trees, which were made to oscillate by pulling on the rope several times; the rope was then released, allowing the tree to perform free oscillations. The case of full crown case and no crown case were examined. In the former case, the trajectory was very thin, while in the latter, radial displacements appeared. James *et al*.^5^ performed extensive measurements in which two strain meters were attached to the trunk, one to measure displacements in the north-south direction, and the one in the east-west direction. The associated software triggered the recording of the displacements, but also of wind speed and directions. This was done for four types of trees: a pole-like tree, a slender tree with branches closely aligned to the trunk, trees with a central trunk structure, and trees with many branches and no central trunk. The authors gave descriptive conclusions about the recorded trajectories: they had a complex pattern, and the third and the fourth type had them always down wind, with some looping and sideways displacements occurring. In the subsequent report^8^, the focus was on the fourth tree type. There was no upwind displacement recorded for this tree in any wind storm event over the 3-month period of monitoring. This general downwind motion from the rest position was found to be similar to some previous reports^9,10^. Kane *et al*.^11^ were interested in large, open-grown trees, and as a result of pull-and-release tests with larger initial displacements, they recorded elliptic-like trajectories of a point on the trunk approximately at 1.4 m above the ground. However, no further insights into these elliptic-like trajectories have been given.

The aim of this work is to develop a theoretical framework for the utilization of the results obtained by using a state-of-the-art infrared marker-tracking system with the focus on the trajectories recorded to determine oscillatory characteristics of the trajectories of the markers arranged along a trunk-dominated tree. The novel procedure emphasizes the importance of the meaning of the shape of the trajectories recorded, which implies the existence of two frequencies calculated, the directions to which they correspond (the so-called, principal axes) and the corresponding damping ratios, which, all in all, give new and precise insights into oscillatory characteristics of out-of-plane vibrating trees, which has not been provided so far.

## Experiments

**Figure 1:**
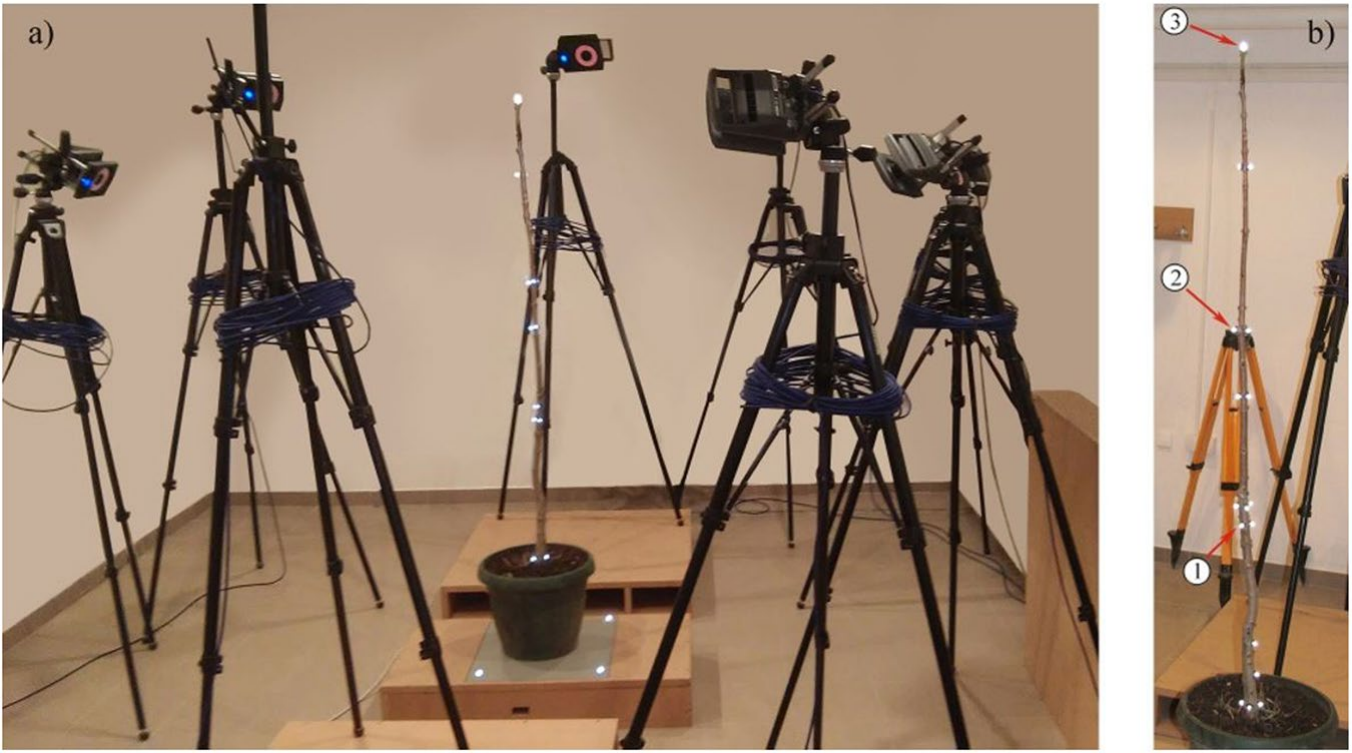
(**a**) The Vicon 3D motion capture system; (**b**) A tree under considerations with several markers along it and three markers numbered (1, 2 and 3).

Experiments were conducted on the de-branched stem of a young pole-like *Aesculisus hippocastanum* tree. This stem was 1540 mm tall with a diameter of 31.5 mm at the base. Pull-and-release tests were carried out, in which the tree was pulled with a rope causing non-zero initial displacements, the system came to the rest state, i.e. initial velocities were zero, and the rope was cut and the tree was released to perform free vibrations. The resulting motions were recorded by a state-of-the-art motion tracking system - Vicon 3D and its accompanying software Nexus 2 (Fig. 1a). This is an infrared (IR) marker-tracking system that offers high resolution of 3D spatial displacements of the reflective markers (dots) arranged along the trunk of the tree. There are eight cameras around the tree and each of them is outfitted with IR optical filters and an array of IR light-emitting diodes (LEDs). The markers reflect the IR radiation emitted by the LEDs, while all other light is filtered so that the system only recognizes the dots. Vicon 3D was originally developed for investigating human motion by visual effects studios, sports therapists, neuroscientists, and for validation and control of computer vision and robotics, but it was originally utilized in this project to track the motion of trees excited in a certain way.

The arrangement of all markers is seen in Fig. 1a,b, but three Markers (1, 2 and 3) are labelled in Fig. 1b as their motion will be analysed subsequently.

**Figure 2:**
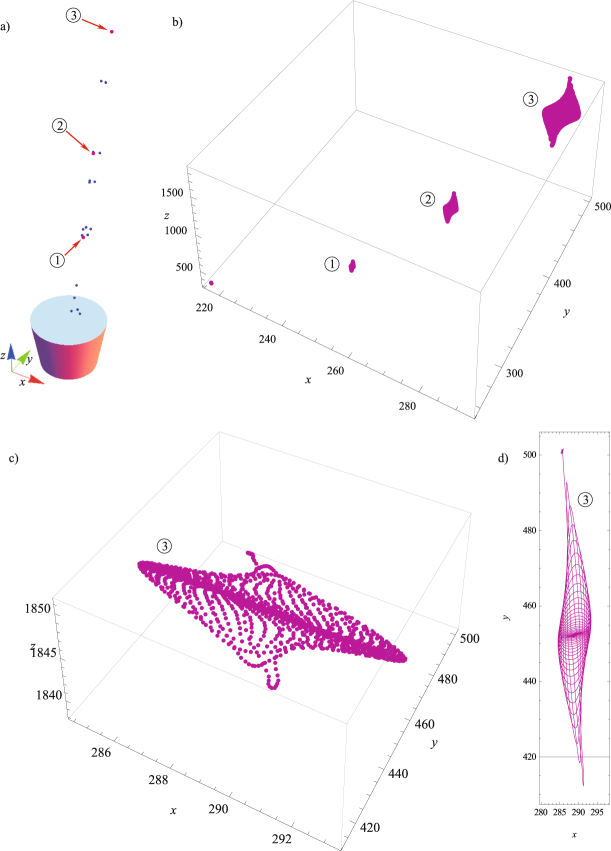
(**a**) The arrangement of all the markers obtained in Wolfram Mathematica and three Markers of interest (1, 2 and 3); (**b**) Trajectories of Markers 1, 2 and 3; (**c**) Trajectory of Markers 3 in 3D; (**d**) Trajectory of Markers 3 in the *x-y* plane.

The 3D displacements recorded are imported in Wolfram Mathematica and then used to construct a three-dimensional representation of the markers, as shown in Fig. 2a. The trajectories of three markers are presented in Fig. 2b. The trajectory of Marker 3 is enlarged and plotted separately in Fig. 2c. Besides presenting the shape of the trajectories, multi-part Fig. 2 also shows the data for the height of the tree and location of Markers 1–3 and their trajectories. Mutual comparisons revealed that the dominant displacements were in the *x*-*y* plane, and the corresponding trajectory is shown in Fig. 2d. The way how this point moves in the *x-y* plane during time is shown in Animation 1, given as a Supplementary file.

The next section is concerned with the development of a theoretical framework that includes the use of these in-plane damped trajectories to obtain the corresponding oscillatory characteristics.

## Theoretical Framework

### Modelling and quantitative insight

**Figure 3:**
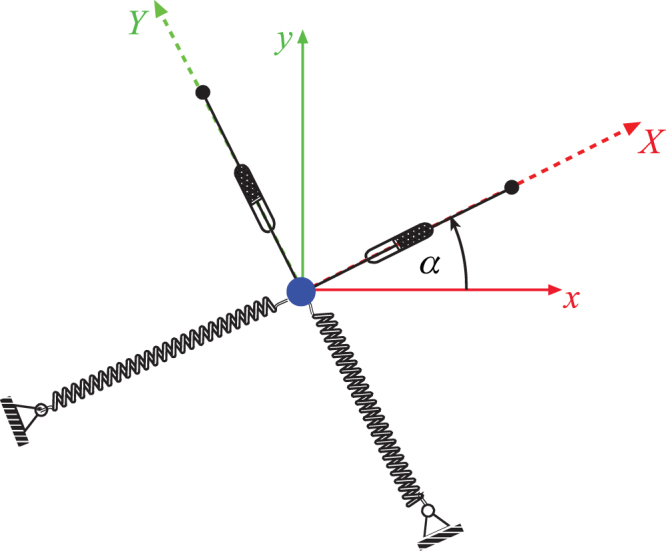
Mechanical model for each marker performing in-plane oscillations.

The mechanical model of each marker performing in-plane vibrations is presented in Fig. 3: it consists of a particle attached to two orthogonal springs^12^, which model elastic properties of the system, and two orthogonal dampers, which include aerodynamic and internal linear viscous damping. Note that the whole system lies in the plane orthogonal to the vertical *z*-axes and that each marker will have its own parameters, but the mechanical and mathematical models used will be the same.

It should be pointed out that there is a possibility for each particle to move along a straight line, but the experiments performed have shown that this does not happen. If the stiffness and damping properties had been the same in all directions, the marker would have oscillated in one direction – the one corresponding to the initial direction, and the system could have been treated as having one degree of freedom. However, the existence of the lateral motion with respect to the initial deflection suggests that there are two degrees of freedom and, thus, two associated natural frequencies *ω*_1_ and *ω*_2_. The directions of the springs are collinear with, the so-called, principal (stiffness) axes, labelled by *X* and *Y* here. Their position is defined by the angle *α* with respect to the coordinate system *x*O*y* used for the measurements and recordings. It is very important to note that, in general, the principal axes differ from the measurement axes.

The differential equations of motion written with respect to the principal axes are:

1 $$\ddot{X}+2{\delta }_{1}\dot{X}+{\omega }_{1}^{2}X=0,$$

2 $$\ddot{Y}+2{\delta }_{2}\dot{Y}+{\omega }_{2}^{2}Y=0,$$

where *δ*_1_ and *δ*_2_ are viscous damping ratios. Note that they are uncoupled. Note also that Equations (1) and (2) do not have time non-dimensionalized, but oscillations take place in real time *t*[s], while the units for other parameters are: *ω*_1_[s^−1^], *ω*_2_[s^−1^], *δ*_1_[s^−1^], *δ*_2_[s^−1^]. These four parameters can be used later on to calculate the damping factors *ζ*_1_ = *δ*_1_/*ω*_1_ and *ζ*_2_ = *δ*_2_/*ω*_2_, which do not have units or can also be expressed in percentages.

**Figure 4:**
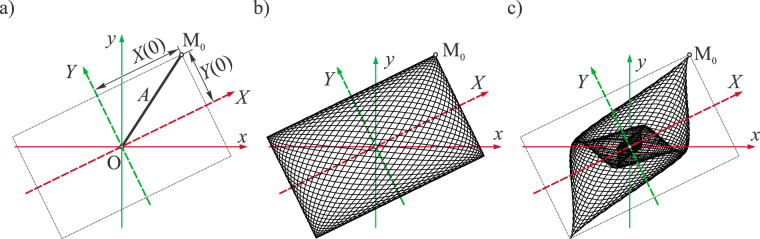
(**a**) Principal axes *X* and *Y*, non-principal axes *x* and *y*, the initial position M_0_, and the rectangular as the envelope of the trajectory; (**b**) Trajectory of undamped motion; (**c**) Trajectory of damped motion.

When the system is conservative (*δ*_1_ = *δ*_2_ = 0) and its motion starts from rest from the position defined by *X*(0) and *Y*(0) (this is the position M_0_ labelled in Fig. 4a), the motion satisfying Equations (1), (2) is given by (Kovacic and Radomirovic, 2017):

3 $$X(t)=X(0)\,\cos ({\omega }_{1}t),\,Y(t)=Y(0)\,\cos ({\omega }_{2}t).$$

Thus, there are the lower and upper boundary for each coordinate: $-X(0)\le X(t)\le X(0)$ and $-Y(0)\le$ $Y(t)\le Y(0)$. Consequently, the trajectory lies inside a rectangle plotted in Fig. 4a. Note that the symmetry axes of the rectangle are parallel to its sides, and coincide with the principal axes. The half-diagonal of the rectangle is defined by the displacement *A* of the particle from the origin. When the motion starts, the particle is in one of the corners. Its velocity, as well as the kinetic energy is zero, while the potential energy is at maximum and it is equal to the overall mechanical energy. Note that in all four corners of the rectangle, the velocity has a zero value as it corresponds to maximal amplitudes. The trajectory fills in the rectangle as time passes, which is illustrated in Fig. 4b.

When the system is non-conservative (${\delta }_{1}\ne 0,{\delta }_{2}\ne 0$), these oscillations become damped and the trajectory narrows down (Fig. 4c). The main questions are how this damped trajectory is related to the rectangle noted and how one can construct the rectangle and use it to obtain the unknown principal axes. To answer these questions, a new procedure is described subsequently.

### How to determine the position of the principal axis

**Figure 5:**
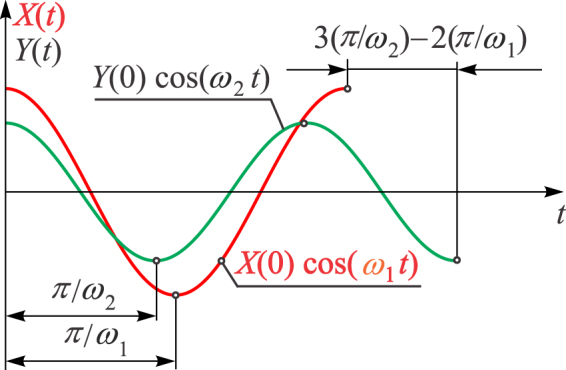
Time-history diagrams along principal axes.

When oscillations start, the particle moves initially almost along the diagonal OM_0_ shown in Fig. 4a. However, as time passes, the lateral motion increases. To achieve the main objective and locate the principal axes, one needs to obtain the moment of time *t*_1_ when the velocity becomes zero, or when it becomes approximately zero. This means that the particle will be in one of the corners of the rectangle or somewhere on the diagonals. To develop a methodology for obtaining *t*_1_, the time history diagrams *X*(*t*) and *Y*(*t*) are considered (Fig. 5).

It is assumed that *ω*_1_ < *ω*_2_ and that the system is undamped. As labelled in Fig. 5, the half-period for *X*(*t*) is ${T}_{X}/2=\pi /{\omega }_{1}$ and the half-period for *Y*(*t*) is ${T}_{Y}/2=\pi /{\omega }_{2}$, where ${T}_{X} > {T}_{Y}$. Note that after each half-period, the velocity in the *X* and *Y* direction is zero. Figure 5 illustrates the situation when three half-periods have been performed along *Y*, while only two have passed for *X*. Generally speaking, the time difference between the number of periods for *Y* and *X* is

4 $$N\frac{\pi }{{\omega }_{2}}-(N-1)\frac{\pi }{{\omega }_{1}} > 0,$$

but it becomes smaller as *N* increases and, the expression on the left-hand side of Equation (4) tends to zero. So, if the number of half-periods for *Y* until *t*_1_ is *n*, one has ${t}_{1}=n\pi /{\omega }_{2}$, while for *X* one holds ${t}_{1}=(n-1)\pi /{\omega }_{1}$. Equating these two expressions leads to

5 $${\omega }_{2}-{\omega }_{1}=\frac{\pi }{{t}_{1}}.$$

**Figure 6:**
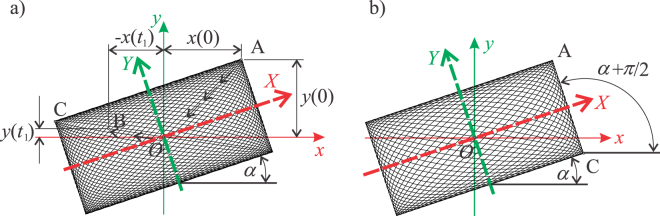
(**a**) Motion of the particle with respect to the characteristic rectangle; (**b**) The case when C is in the right lower angle.

If *n* is an even integer, the particle reaches the corner C at *t*_1_ labelled in Fig. 6a, because the number of half-periods for *Y* is even and for *X* odd.

If *n* is an odd integer, the particle reaches the corner C at *t*_1_ and its position is shown in Fig. 6b. Note that the number of half-periods for *Y* is odd then, while it is even for *X*.

Of interest is also the case when *n* is not an integer. In this case, the particle is not in the corner at the moment *t*_1_, but somewhere on the diagonal OC, and this position is labelled by B in Fig. 6a. At the moment *t*_1,_ the rectangle as the envelope of the trajectory is completed. From *t*_1_ to 2*t*_1_, the particle forms the same rectangle, and it is again located on the diagonal OA. However, at 5*t*_1_ the same rectangle is again formed for the fifth time, but the particle is on the diagonal OC. When the system is non-conservative (*δ*_1_≠0 and *δ*_2_≠0), there is no rectangle, but a shape shown in Fig. 4c occurs. During the time interval $0\le t\le {t}_{1}$, the first external figure is formed. Then, another form is composed inside it, as seen in Fig. 4c. When the damping ratios are equal and small, the rounded corners of these figures lie on the diagonals of the rectangle.

To deal with the damped case, the analysis procedure is analogous to the one for the undamped case, but instead of half-periods, one deals with quasi-half-periods and damped frequencies $\,{\bar{\omega }}_{1}=\sqrt{{\omega }_{1}^{2}-{\delta }_{1}^{2}}$ and ${\bar{\omega }}_{2}=\sqrt{{\omega }_{2}^{2}-{\delta }_{2}^{2}}$, i.e.

6 $${T}_{X}/2=\frac{\pi }{{\bar{\omega }}_{1}}=\frac{\pi }{\sqrt{{{\omega }_{1}}^{2}-{{\delta }_{1}}^{2}}},\,{T}_{Y}/2=\frac{\pi }{{\bar{\omega }}_{2}}=\frac{\pi }{\sqrt{{{\omega }_{2}}^{2}-{{\delta }_{2}}^{2}}}.$$

Equation (5) now becomes

7 $${\bar{\omega }}_{2}-{\bar{\omega }}_{1}=\frac{\pi }{{t}_{1}}$$

Thus, if one finds the moment of time *t*_1_ when the velocity is zero, one can easily calculate the difference between the damped frequencies by using Equation (7).

**Figure 7:**
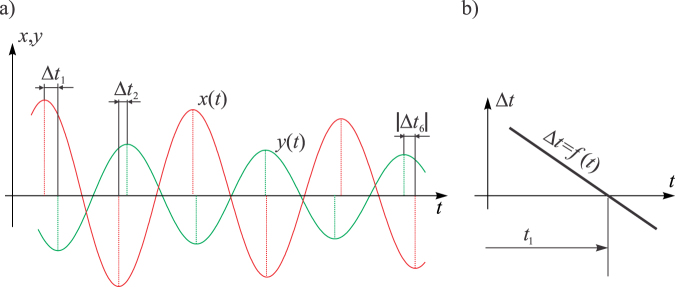
(**a**) Time differences between the extrema of *x*(*t*) and *y*(*t*); (**b**) Time difference versus time diagram with its zero *t*_1_.

Let us show now the procedure for determining *t*_1._ To that end, one should recall that each period or half-periods for the principal coordinates starts and ends up with the respective velocity being zero and *X*(*t*) and *Y*(*t*) being extremal. The same fact holds for quasi-periods or half-periods for *x*(*t*) and *y*(*t*). This implies that one needs to consider the extrema of the time-histories of the recorded *x*(*t*) and *y*(*t*) as plotted in Fig. 7a and the time differences between them (they are labelled by Δ*t*_1_, Δ*t*_2_… in Fig. 7a). If *x*(*t*) and *y*(*t*) would have extremal values simultaneously, the corresponding moment of time would correspond to *t*_1._ However, due to the existence of damping, this is highly unlikely, and one should actually represent the differences from Fig. 7a as the function of time and find its zero (Fig. 7b).

Let us show now how to obtain the angle *α*. Referring to Fig. 6a, the vectors **OA** and **OB** can be expressed as:

8 $${\bf{OA}}=x(0){\bf{i}}+y(0){\bf{j}},$$

9 $${\bf{OB}}=x({t}_{1}){\bf{i}}+y({t}_{1}){\bf{j}}.$$

One can then notice that the vector **OC** and **OB** are collinear, and can be related as follows

10 $${\bf{OC}}=\lambda \,{\bf{OB}}=\lambda [x({t}_{1}){\bf{i}}+y({t}_{1}){\bf{j}}],$$

where *λ* is the parameter of proportionality. Based on the equality of diagonal of the rectangle, this parameter can be expressed as

11 $$\lambda =\frac{\overline{OC}}{\overline{OB}}=\frac{\overline{OA}}{\overline{OB}}=\frac{\sqrt{x{(0)}^{2}+y{(0)}^{2}}}{\sqrt{x{({t}_{1})}^{2}+y{({t}_{1})}^{2}}}.$$

The angle *α* is now defined by

12 $$\cos \,\alpha =\frac{{\bf{CA}}\cdot {\bf{i}}}{\overline{CA}}=\frac{x(0)-\lambda x({t}_{1})}{\sqrt{{[x(0)-\lambda x({t}_{1})]}^{2}+{[y(0)-\lambda y({t}_{1})]}^{2}}}.$$

So, after calculating *t*_1_, one should determine *x*(*t*_1_) and *y*(*t*_1_) from the experimentally recorded time histories. Then, by using Equation (11) one calculates first the parameter *λ*, and then the angle *α* from Equation (12). If the position of C is as shown in Fig. 6b, one has

13 $$\cos (\frac{\pi }{2}+\alpha )=\frac{{\bf{CA}}\cdot {\bf{i}}}{\overline{CA}}=\frac{x(0)-\lambda x({t}_{1})}{\sqrt{{[x(0)-\lambda x({t}_{1})]}^{2}+{[y(0)-\lambda y({t}_{1})]}^{2}}}.$$

### How to determine natural frequencies and damping ratios

Now, when the angle *α* is known, one can pass from the non-principal axes to the principal axes by using the following coordinate transformations (see Fig. 3):

14 $$X=x\,\cos (\alpha )+y\,\sin (\alpha ),$$

15 $$Y=-\,x\,\sin (\alpha )+y\,\cos (\alpha ).$$

The solutions of motion with respect to the each of the principal axes are (Kovacic and Radomirovic, 2017):

16 $$X(t)={{e}}^{-{\delta }_{1}t}\,\{X(0)\cos ({\bar{\omega }}_{1}t)+X(0)\frac{{\delta }_{1}}{{\bar{\omega }}_{1}}\,\sin ({\bar{\omega }}_{1}t)\},$$

17 $$Y(t)={{e}}^{-{\delta }_{2}t}\{Y(0)\cos ({\bar{\omega }}_{2}t)+Y(0)\frac{{\delta }_{2}}{{\bar{\omega }}_{2}}\,\sin ({\bar{\omega }}_{2}t)\}.$$

A numerical fitting procedure with these two analytical expressions gives the values of the damping ratios, damped frequencies, and consequently two natural frequencies.

## Numerical analysis and parameter values

**Figure 8:**
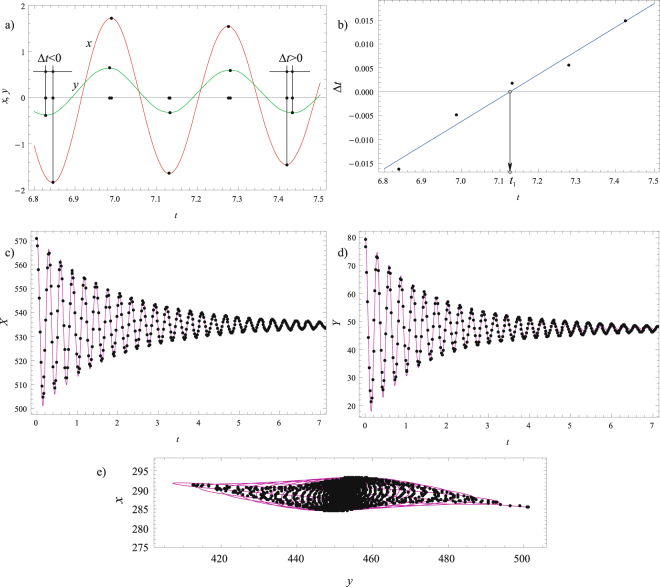
Marker 3: (**a**) Time differences between the extrema of *x*(*t*) and *y*(*t*); (**b**) Time difference versus time diagram with its zero value *t*_1_; (**c**) Comparison of the transformed experimental results (magenta solid line) and the fitted numerical results (black dots) along the *X*-axis; (**d**) Comparison of the transformed experimental results (magenta solid line) and the fitted numerical results (black dots) along the *Y*-axis; (**e**) Comparisons for the trajectories.

In accordance with the previously presented procedure, the recorder time histories for the non-principal axes *x* and *y* are considered. Marker 3 is chosen to start with, and the corresponding time differences between the extrema are shown in Fig. 8a, while the graph ‘time differences versus time’ is plotted in Fig. 8b. Its zero corresponds to the value of *t*_1,_ which is also presented in Table 1. The angle *α* is calculated by using Equation (12) and it is also included in Table 1. The analytical expressions (16) and (17) are used in conjunction with the in-built FindFit method to determine the damping ratios and two natural frequencies, which are included in Table 1 as well. The comparisons between the transformed experimental results along principal axes, as well as between the trajectories are given in Fig. 8c–e. As can be seen, the agreements achieved are very good. It should also be pointed out that similar damped time histories and trajectories have been reported on real open-grown trees^13^, which supports the results presented herein and the applicability to real conditions.

**Table 1:** Values of the calculation and system parameters obtained numerically based on the analytical procedure developed.

Parameter values							
Marker		${{\boldsymbol{t}}}_{{\bf{1}}}$ $[{\boldsymbol{s}}]$	${\boldsymbol{\alpha }}[{\boldsymbol{^\circ }}]$	${{\boldsymbol{\omega }}}_{{\bf{2}}}[{{\bf{s}}}^{{\boldsymbol{-}}{\bf{1}}}]$	${{\boldsymbol{\omega }}}_{{\bf{2}}}[{{\bf{s}}}^{{\boldsymbol{-}}{\bf{1}}}]$	${{\boldsymbol{\delta }}}_{{\bf{1}}}[{{\bf{s}}}^{{\boldsymbol{-}}{\bf{1}}}]$	${{\boldsymbol{\delta }}}_{{\bf{2}}}[{{\bf{s}}}^{{\boldsymbol{-}}{\bf{1}}}]$
1	*X*	7.086	52.524	21.557		0.517	
	*Y*				21.839		0.65
2	*X*	7.115	52.954	21.563		0.525	
	*Y*				21.858		0.654
3	*X*	7.126	52.376	21.564		0.518	
	*Y*				21.862		0.642

**Figure 9:**
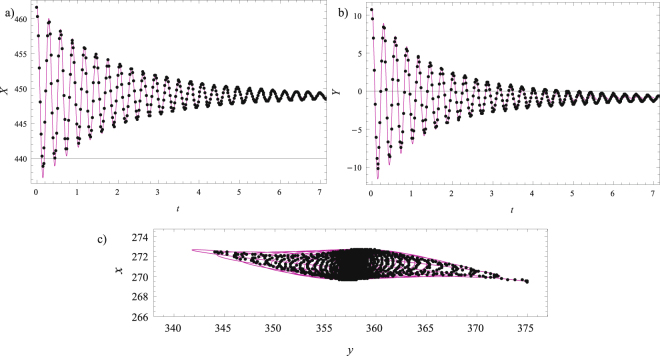
Marker 2: (**a**) Comparison of the transformed experimental results (magenta solid line) and the fitted numerical results (black dots) along the *X*-axis; (**b**) Comparison of the transformed experimental results (magenta solid line) and the fitted numerical results (black dots) along the *Y*-axis; (**c**) Comparisons for the trajectories.

**Figure 10:**
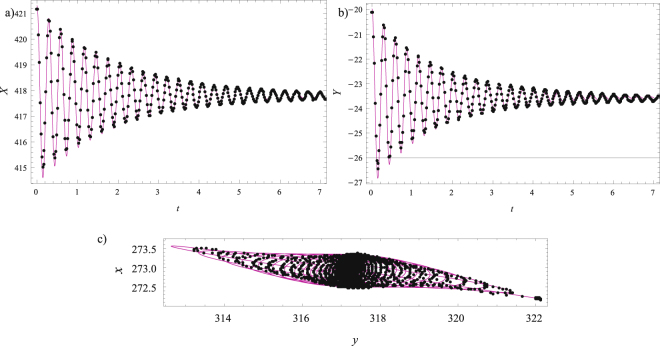
Marker 1: (**a**) Comparison of the transformed experimental results (magenta solid line) and the fitted numerical results (black dots) along the *X*-axis; (**b**) Comparison of the transformed experimental results (magenta solid line) and the fitted numerical results (black dots) along the *Y*-axis; (**c**) Comparisons for the trajectories.

To whole procedure is repeated for Markers 2 and 1, and the values of the corresponding parameters are included in Table 1. The confirmations of good agreement between the transformed experimental results along principal axes and the trajectories are illustrated in Fig. 9 for Marker 2 and in Fig. 10 for Marker 1.

Analysing Table 1, one finds that this procedure gives that the position of principal axes along the trunk is between 52° and 53°. The natural frequencies have close values, which are approximately *ω*_1_ = 21.6 s^−1^ and *ω*_2_ = 21.86 s^−1^. This yields the frequency of vibration being approximately *f*_1_ = 3.44 Hz and *f*_2_ = 3.48 Hz, respectively. The damping ratios corresponding to the directions of two principal axes are, approximately, 0.52 s^−1^ and 0.65 s^−1^. These values imply the following values of the damping factor *ζ*_1_ = *δ*_1_/*ω*_1_ = 0.024 and *ζ*_2_ = *δ*_2_/*ω*_2_ = 0.03 (or, 2.4% and 3%). The damping factors are seen to be small as no branches and leaves were present, which contribute to their higher values^14^.

## Qualitative insight

**Figure 11:**
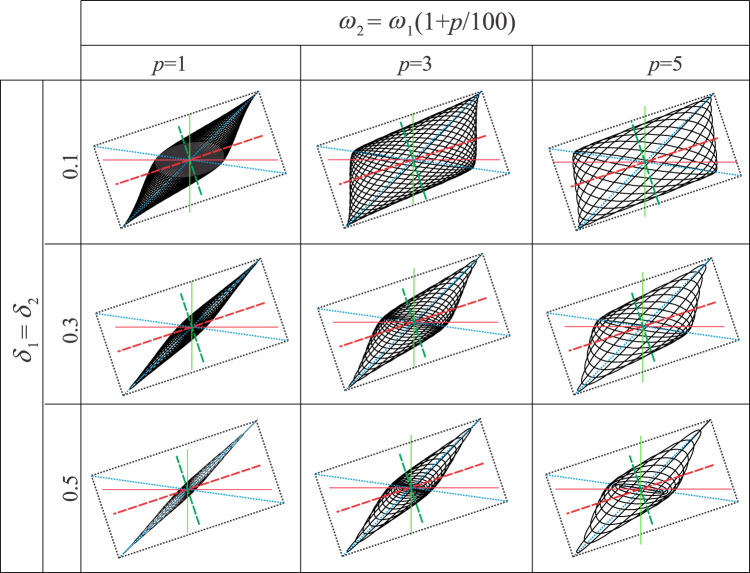
Shapes of trajectories for different percentage differences between the natural frequencies and various values of the damping ratios (note that its units are s^−1^). The position of the horizontal and vertical (measurement) axes is noted as well as the position of the principal axes and the sides of the rectangle defined.

The experiments showed that the values of *ω*_1_ and *ω*_2_ are close to each other, with the difference of around 1%. Therefore, the values of *ω*_1_ and *ω*_2_ differ slightly and can be related to each other in the form ${\omega }_{2}={\omega }_{1}(1+p/100)$, i.e. the parameter *p* can be introduced as their difference in percentages. The aim of this section is to present how the shape of the trajectory changes when this percentage increases as well as when the damping ratio varies. It is expected that these trajectories and their shape can be used to gain a qualitative insight into the closeness of the natural frequencies and the value of the damping ratios. It is assumed that *ω*_1_ = 20 s^−1^, while *p* is taken to be 1%, 3% and 5%. The viscous damping ratios are assumed to be of the increasing values 0.1 s^−1^; 0.3 s^−1^ and 0.5 s^−1^. All the corresponding trajectories are presented in Fig. 11, together with the principal stiffness axes, measurement axes and the sides of the rectangle (the same colour and style legend is used for the axes as in Fig. 4) to emphasize how the shape and the position of the trajectory are related to their position.

All these cases enable one to gain the following insights into the oscillatory characteristics based on the shape of trajectories: the lower the value of *p*, the narrower the trajectories; the trajectories become narrower also when the damping increases. Thus, going back to the experimentally obtained trajectory given in Fig. 2d, one can conclude that this trajectory is narrow, and, therefore, comparable to some of those from Table 1 corresponding to the smaller *p* and smaller damping ratios.

## Conclusions

This study has been concerned with a completely new and original utilisation of the trajectory of markers arranged along a trunk-dominated (de-branched, pole-like) potted tree that performs free vibrations. This includes both quantitative and qualitative insights into their in-plane motion recorded with a view to obtaining the corresponding fundamental and precise oscillatory characteristics that have not been determined so far. A high-tech motion-tracking system has been used to record displacements of the markers arranged along the tree under consideration.

As the markers (particles) from the trunk have been found to move approximately in a plane parallel to the horizontal plane, the mechanical model for describing their motion has two degrees of freedom. It has been introduced herein as consisting of two orthogonal springs and dampers, the position of which is defined by two principal axes. The experimental results are analysed quantitatively based on this mechanical model, giving the position of the principal axes. As far as the authors are aware, neither the principal axes nor the utilisation of the trajectory for their determination has been presented so far related to this type of trajectories and tree vibrations. In the case studied, one of the principal stiffness axes has been found to be located under the angle ranging between 52° and 53° with respect to the horizontal measurement axis. The other principal axis is orthogonal to it. There are two natural frequencies associated with each of these principal axes and they have close values: one is calculated to be approximately 21.6 s^−1^ and the other one 21.86 s^−1^ (the former is approximately 3.44 Hz and the latter is slightly higher than it). The damping ratios related to the directions of principal axes are, approximately, 0.52 s^−1^ and 0.65 s^−1^, which correspond to approximate damping factors of, respectively, 0.024 and 0.03.

The approach focused on trajectories presented herein also offers the possibility to estimate the difference between two natural frequencies qualitatively, without any calculations, and to estimate how small/large the overall damping is. This can be done based on the shape of trajectories. Thus, a thinner trajectory implies that the difference between two natural frequencies is smaller as well as that damping is larger.

The knowledge of the position of the principal axes when a tree does not perform in-plane oscillations is important both for the characterization of its structural elastic properties as well as for experiments: pulling a tree initially in the direction of one of them and releasing it would make the point on the tree oscillate along a straight line with one of the natural frequencies; similarly, pulling a tree initially in the orthogonal direction and releasing it would make this point oscillate along the orthogonal straight line with the other natural frequency. Applying the pull-and-release test in an arbitrary direction between the principal axes will cause a tree oscillate with a frequency that is a certain combination of these two natural frequencies, and this depends on the direction of pulling. It should be noted that in some previous experiments with trees in the natural environment, two-closed valued frequencies in two orthogonal directions have been obtained: in the north-south and east-west directions^11^, or in the fall-line and cross-slope direction^15^. Yet, there has not been any validation that these directions corresponded to their principal axes. Contemporary high-tech motion capture systems with multiple cameras offer the possibilities for very detailed and precise data in different motion analysis applications, and the study presented herein has provided the related proof-of-concept for its use for recording motion of points on a tree. Being accompanied with the new theoretical methodologies, this study has revealed some primary research details about trees that do not perform in-plane oscillations.

So, three complementary approached have been used in this work: a high-tech experimental technique, a new analytical and contemporary numerical approaches, enabling one to gain a detailed and very prices insight into dynamics and oscillatory properties of the object under consideration. This work opens up several avenues for progress regarding the use of this high-tech motion capture system –on indoor potted trees, or on open-grown trees. First, branched trees should be equipped with markers, placed on the points on branches of different branching hierarchy and also on different locations on the same branching order to determine their trajectories and oscillatory characteristics. Second, the markers are (dynamically speaking) treated as particles performing small vibrations in one plane, so each of them represents a linear system with two degrees of freedom and, thus, have two natural frequencies. However, the markers/particles on branches might move in 3D and have three degrees of freedom and three natural frequencies associated with three principal axes. In addition, real trees are continuous systems, i.e. systems with an infinite number of degrees of freedom and have an infinite number of modes and modal frequencies. To catch this property, markers could be arranged along a trunk very close to each other (or completely continuously) and further on along branches. This could yield valuable details about deformations of certain parts of the tree (bending and torsion). Last but not least, trees can be pulled to perform large-amplitude vibrations and this could be realised with different initial amplitudes, which will bring geometric nonlinearity to bear. The question of interest is if the natural frequencies would be changing with the amplitude as in nonlinear oscillators^16^ or if they will be amplitude-independent, behaving thus as nonlinear isochronous systems^17^. All these open questions and their answers would help theoretical biologist advance their knowledge on tree biomechanics related to their oscillatory properties. It is also expected that the precise determination of these oscillatory properties will be beneficial to forestry and arboriculture, helping to assess the structural integrity and risk assessments of certain branches or the whole tree.

The trajectories recorded and characterised have been shown to stem from 2D damped orthogonal oscillations. They were recorder on trees, and their explanation and characterisation have yielded new insights into the related oscillatory characteristics, which can be further used in the closely related fields when trees perform out-of-plane vibrations: arboriculture, forestry and botany, especially related to the closeness of the natural frequency and the position of the principal axes. However, orthogonal oscillations appear or are associated with disparate systems that occur in many other fields and devices: optics, electronics and electrical engineering, material characterisation, harmonographs (devices containing coupled pendula), etc. It is, therefore, expected that specialists from other disciplines will benefit from the methodologies presented herein as well.

## Electronic supplementary material

Supplementary file
