# Supplementary figures and images for: Characterisation of tree vibrations based on the model of orthogonal oscillations
Source: Sci Rep. 2018 Jun 4;8:8558. doi: 10.1038/s41598-018-26726-5 (PMC5986778; doi:10.1038/s41598-018-26726-5)

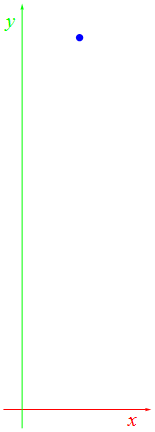

Supplement: Supplementary file 1 — Supplementary file [file 41598_2018_26726_MOESM1_ESM.gif]
